# Dataset on some soil properties improvement by the addition of olive pomace

**DOI:** 10.1016/j.dib.2019.103878

**Published:** 2019-03-28

**Authors:** Adnan I. Khdair, Sawsan I. Khdair, Ghaida A. Abu-Rumman

**Affiliations:** a Jordan University of Science and Technology (JUST), P.O.Box: 3030, Irbid 22110, Jordan; b King Abdulaziz University, College of Engineering, P.O.Box: 80204, Jeddah 21589, Saudi Arabia; c Faculty of Pharmacy, Al-Zaytoonah University of Jordan, 11733 Amman, Jordan; d Department of` Civil Engineering, Isra University, 11622 Amman, Jordan

## Abstract

Soil amendment with olive cake produced from olive mills waste (olive pomace/cake) is an ordinary practice in olive producing countries in the Middle East. It is used to improve soil physical and chemical properties as well as cheep waste management approach. But, the olive cake contains small percentage of residual oil which may affect water holding capacity of soil and penetration rate in agricultural lands. The data provided in this article shows the influence of adding olive pomace to clay and sand clay soils in terms of water holding capacity (WHC), penetration depth and accumulate intake.

**Table:** Specifications table

Subject area	*Agricultural and Biological Sciences*
More specific subject area	*Soil Science*
Type of data	*Table, graph, figure*
How data was acquired	*Laboratory measurements using pressure plate apparatus model number: 0750 SAIF*[1]*to draw soil water retention curves (SWR); The FEL5 Demonstration Infiltration Apparatus – Issue 1 was used for infiltration tests*[2]
Data format	*Raw, analyzed*
Experimental factors	*Soil samples were dried and screen through 2-mm strainer while the olive cake freeze dried and ground to pass through 1 mm sieve*
Experimental features	*The effect of olive cake addition on clay and sand clay soil were investigated. Soil parameter studied were soil water retention curves (SWR), infiltration and water holding capacity (WHC) at olive cake addition of 3%, 6% and 9% by weight. A pressure plate apparatus was used to obtain soil water retention curves at a pressure range from 0.30 (wilting) to 1500 kPa (Saturation). FEL5 Demonstration Infiltration Apparatus*[2]*with three gradual perspex cylinders were used for penetration depth measurements.*
Data source location	*Amman, Jordan, Latitude (°N) 29′33′, Longitude (°E) 35′00′, Elevation 772 m.*
Data accessibility	*The data is included in this article.*
Related research article	*Abu-Rumman Ghaida, Effect of Olive Mill Solid Waste on Soil Physical Properties. International journal of Soil Science. 2016, 11(3): pp. 94–101.*10.3923/ijss.2016.94.101[3]

**Table:** 

**Value of the data** • The data showed that the addition of olive cake to soil improves soil properties such as: water holding capacity (WHC) and accumulation intake important factors for plant growth [3], [4]. • Clay soils have larger surface area compared to sandy soil; therefore holding more water at higher tensions near the wilting point. • Soils in semi-arid and arid regions are poor in organic matter as a result of desertification; the addition of olive cake to soil will increase soil fertility and penetration depth. • The addition of organic matter reduce soil bulk density as reported in literature [5] which might reduce soils erosion as a result of soil aggregation improvement. • The dataset may serve as a benchmark for future studies on the effect of olive pomace addition as soil amendments on other soils properties.

## 1 Data

**Fig. 1:**
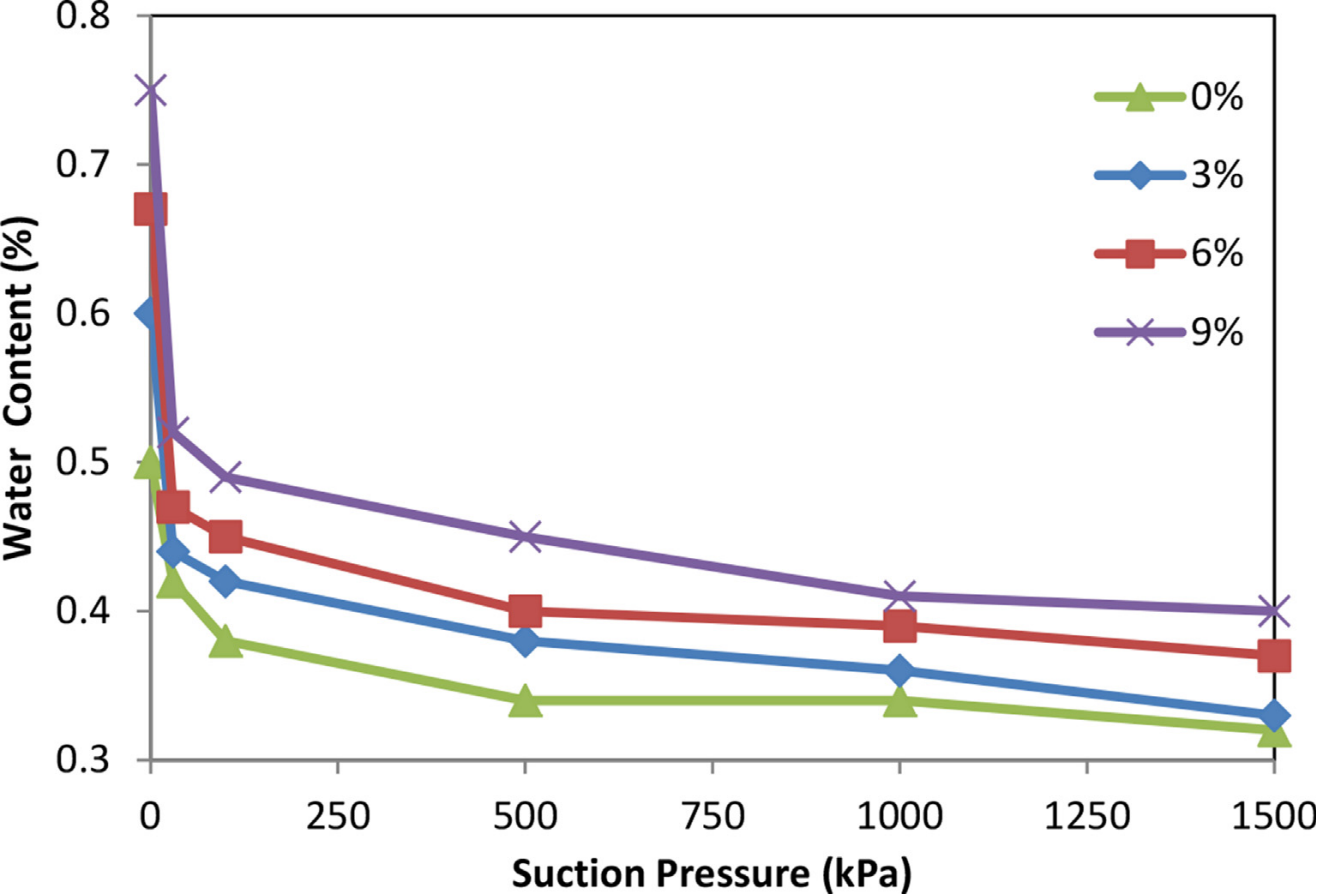
Soil-Water-retention curves for clay soil as affected by olive cake.

**Fig. 2:**
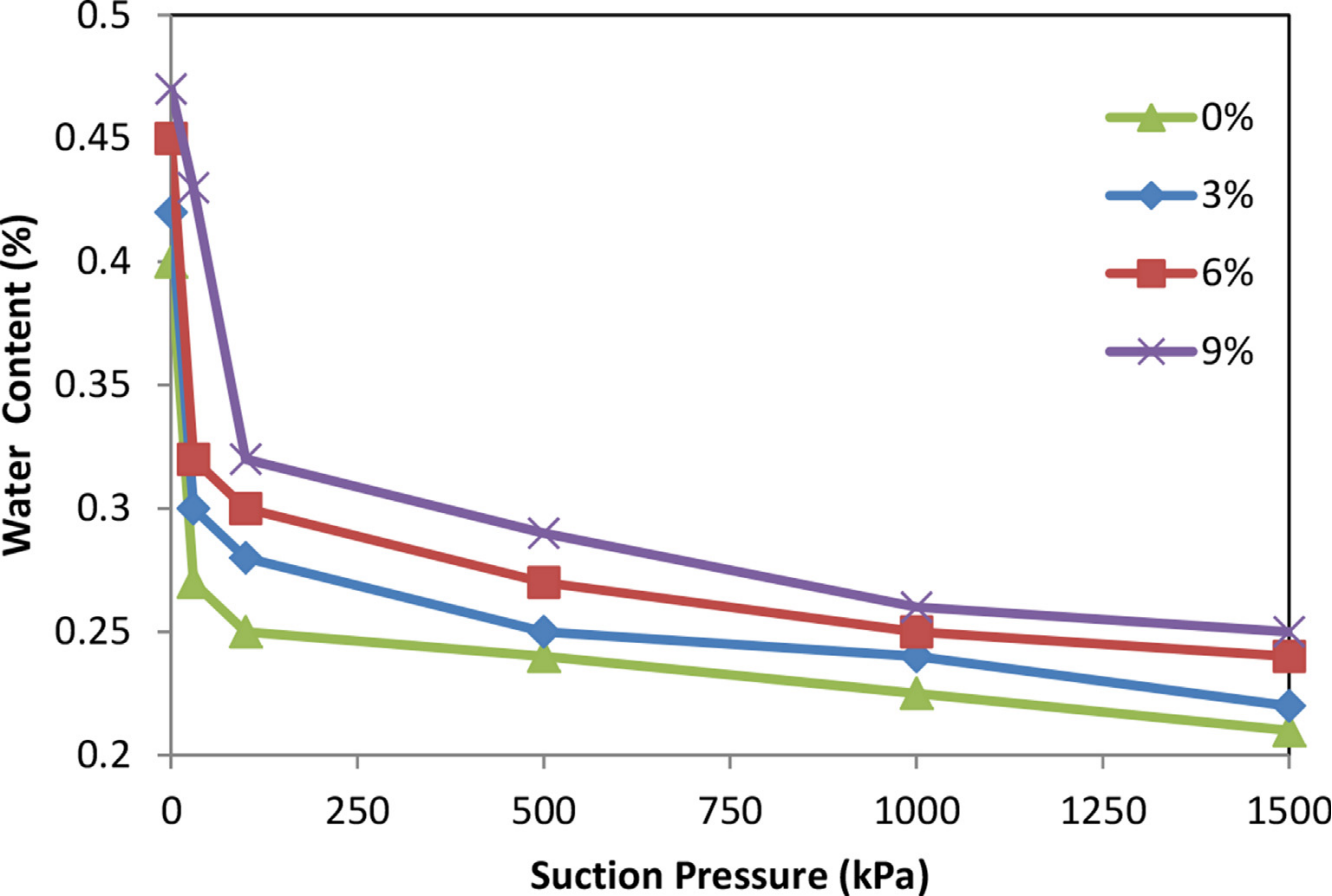
Soil-Water-retention curves for sandy clay soil as affected by olive cake.

The soil water retention curves (SWR) are shown in Fig. 1 for clay soil (20% sand, 25% silt, 55% clay) and in Fig. 2 for sandy clay soil (55% sand, 5% silt, 40% clay) as affected by olive cake addition. The water holding capacity (WHC) increased as olive cake application rates increased compare to the control in agreement with [4]. Clay soils hold more water at higher tensions compared to sandy soils because they have larger surface area. This in agreement with [5], who reviewed the effects of organic matter addition as soil amendment for many soils with different texture textures.

The penetration depths were read directly from the three transparent gradual scales cylinders (FEL5 Demonstration Infiltration Apparatus [2])*.* The penetration depths are shown in Table 1 for clay soil and Table 2 for sandy clay soil as affected by olive cake addition. The data shows that penetration depth increased as olive cake application rate increased. The addition of olive cake increased the soil organic content, which favor large soil aggregates formations, hence resulted in larger penetration depth [3], [5].

**Table 1:** Penetration depth (mm) as affected by olive pomace addition for clay soil.

Time (min)	Pomace application rate			
	0%	3%	6%	9%
0	0	0	0	0
2	25	30	35	40
5	35	40	50	60
7	55	60	70	75
9	65	70	80	83
12	80	85	100	105
15	90	100	120	125
20	115	130	145	150
24	125	135	170	180
34	135	150	183	195
45	139	157	200	210
50	145	165	210	223
75	152	175	220	233
100	154	180	225	240
125	157	190	235	250
150	160	200	250	265
200	165	215	265	280

**Table 2:** Penetration depth (mm) as affected by olive pomace addition for sandy clay soil.

Time (min)	Pomace application rate			
	0%	3%	6%	9%
0	0	0	0	0
3	10	11	50	50
6	15	16	90	90
9	30	32	130	125
12	50	52	150	140
15	70	73	175	160
18	85	90	185	170
21	105	110	200	185
24	120	130	175	170
34	128	140	200	210
45	139	155	230	265
50	150	160	250	240
75	152	170	275	300
100	154	175	305	320
125	157	180		
150	160	187		
200	165	192		

The water accumulate intake (mL) is shown in Table 3 for clay soil and Table 4 for sandy clay soil as affected by addition of olive cake. The data shows that accumulate intake increased as olive pomace application rate increased, this in agreement with [3], [4], [6], [7].

**Table 3:** Accumulated intake (mL) as affected by olive pomace addition for clay soil.

Time (min)	Pomace application rate			
	0%	3%	6%	9%
0	0	0	0	0
2	15	10	12	14
4	30	20	23	26
6	35	27	29	31
10	40	33	35	37
12	45	38	49	43
15	50	45	47	49
22	57	50	53	55
25	62	53	57	60
35	63	60	65	70
40	64	65	70	75
50	65	73	80	85
75	65	82	90	95
100	65	93	99	105
150	65	100	107	112
200	65	100	105	110

**Table 4:** Accumulated intake (mL) as affected by olive pomace addition for sandy clay soil.

Time (min)	Pomace application rate			
	0%	3%	6%	9%
0	0	0	0	0
3	4	5	15	20
6	7	9	20	25
9	10	13	25	33
12	13	17	35	48
15	16	19	40	57
18	19	22	50	65
21	23	26	60	75
25	25	32	70	80
34	33	35	78	85
45	43	45	85	90
50	47	50	90	95
75	50	53	100	105
100	58	65	112	113
125	63	70	110	111
150	65	78	110	111
200	65	75	110	111

Normally, the clayey soil has less penetration and water intake than sandy clay soil as shown in Table 5, this in agreement with [5], [8], [9].

**Table 5:** Penetration depth and accumulated intake with (time).

	Clay			Sandy clay				
	0%	3%	6%	9%	0%	3%	6%	9%
Penetration depth (mm)	160 (24 hr)	210 (24 hr)	290 (24 hr)	300 (24 hr)	180 (24 hr)	280 (24 hr)	305 (100 m)	320 (100 m)
Accumulated intake (ml)	69 (24 hr)	95 (24 hr)	107 (150 m)	112 (150 m)	63 (24 hr)	78 (150 m)	112 (100 m)	113 (100 m)

**Table 6:** Physico-chemical properties of olive pomace.

Property	
Moisture content	60.90
pH	5.25
Organic C	620
Total N (g/kg)	2.4
Total P (g/kg)	0.65
Total K (g/kg)	1.05
Carbon/Nitrogen (C/N)	30.50
Ash (g/kg)	75.3

## 2 Experimental design, materials, and methods

**Fig. 3:**
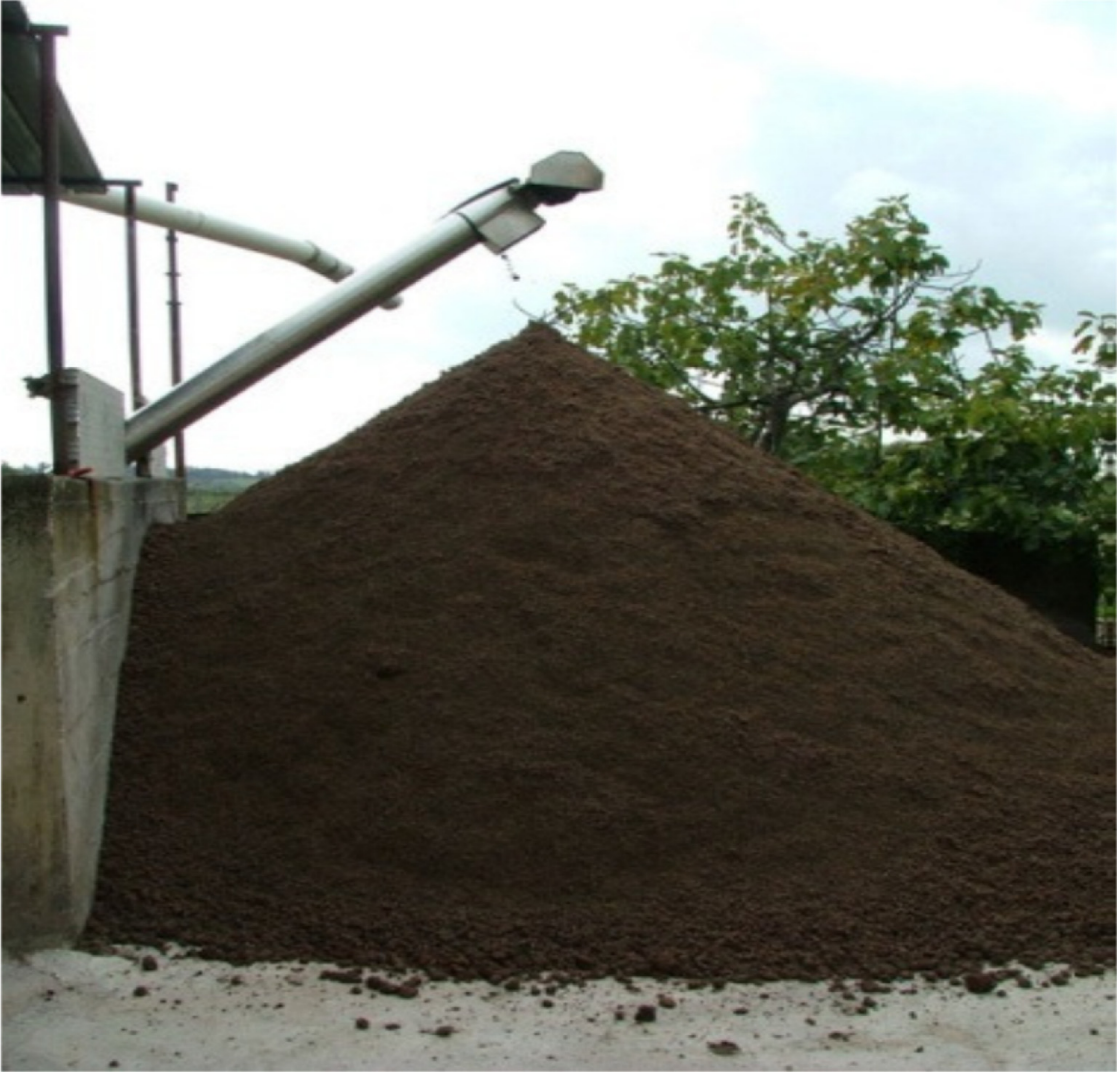
Olive pomac/cake.

Olive pomace addition on soil water holding capacity, penetration depth and accumulated intake were examined for clay and sandy clay Soils. The soils samples obtained from the top soils surfaces, crushed dried and passed through a 2 mm strainer to remove bulky fragments. The olive cake is shown in Fig. 3 which was obtained from a three phase olive mill, freeze-dried and ground to pass through a 1 mm sieve. Several tests were done for each soil at three olive cake application rates (3%, 6% and 9%) on dry weight basis in addition to 0% the control. For each test olive cake was added to the soil sample and mixed thoroughly in plastic bag before used. The penetration depths were read directly from three transparent gradual scales cylinders (FEL5 Demonstration Infiltration Apparatus [2]).

The olive pomace physicochemical properties are show in Table 6, which include moisture content, pH, organic C, N—P—K, (C/N) and ash.

Soil-water-retention curves were obtained according to Ref. [1] by pressure plate apparatus from welting pressure 0.30 to saturation pressures 1500 kPa, using 70-mm diameter PVC rings. The soil samples were saturate by distilled water. The soil moisture contents were evaluated at different suction pressure 30–1500 kPa. The SWR curves constructed for each soil represent the averages of the pressure plate tests from all rings.

### 2.1 Infiltration (penetration depth)

FEL5 Demonstration Infiltration Apparatus – Issue 1 [2] used for infiltration measurements. Soil samples were mixed thoroughly and filled gradually to avoid segregation of soil particles in cylinders up to 380 mm mark in the apparatus. Water discharge was collected by 500 mL beakers placed below the three infiltration cylinders. The initial soil and water surfaces were marked. Each cylinder received equal head of water of 100 mL at the same time. As the wetting frontage advanced, the differences between water and soil surface level were recorded at time intervals of (1, 3, 5, 7, 9, 20, 35, 47, 60, 75, 90, 110, 170, 250 min and after 24 hr). The accumulated water intake in the soil was determined using the following relations:

(1) $${\text{I}}_{\text{D}}={\text{I}}_{\text{W}}-{\text{I}}_{\text{S}}$$

(2) $${\text{H}}_{\text{D}}={\text{H}}_{\text{W}}-{\text{H}}_{\text{S}}$$

(3) $${\text{A}}_{\text{I}}={\text{I}}_{\text{D}}-{\text{H}}_{\text{D}}$$

where I_D_ is the initial depth, I_W_ and I_S_ are the initial water and soil surface heights, respectively. H_D_ is water depth as time elapsed and H_W_ and H_S_ are the heights of water and soil surface, receptively. A_I_ is the accumulated intake calculated from Eq. (3) after the data collected throughout the tests.

## References

[1] ASTM D2325-68 (2000). Standard Test Method for Capillary-Moisture Relationships for Coarse- and Medium-Textured Soils by Porous-Plate Apparatus.

[2] Armfield Group (2012). Armfield Technical Education Company FEL5 Demonstration Infiltration Apparatus – Issue 1.

[3] Abu-Rumman G. (2016). Effect of olive mill solid waste on soil physical properties. Int. J. Soil Sci..

[4] Abu-Zreig M., Al-Widyan M. (2005). Influence of olive mills solid waste on soil hydraulic properties. Commun. Soil Sci. Plant Anal..

[5] Khaleel R., Reddy K.R., Overcash M.R. (1981). Changes in soil physical properties due to organic waste applications: a review. J. Environ. Qual..

[6] El-Asswad R.M., Said A.O., Mornag M.T. (1993). Effect of olive oil cake on water holding capacity of sandy soils in Libya. J. Arid Environ..

[7] Al-Widyan M., Al-Abed N., Al-Jaleel H. (2005). Effect of composted olive cake on soil physical properties. Commun. Soil Sci. Plant Anal..

[8] Giovanna C., Giovanni L., Leonardo C. (2008). Improvement of soil properties by application of olive oil waste. Agron. Sustain. Dev..

[9] Barbera A.C., Maucien C., Cavallaro V., Loppolo A., Spagna G. (2013). Effects of spreading olive mill wastewater on soil properties and crops, a review. Agric. Water Manag..

